# Rapid ventricular overdrive pacing and other advanced flow-control techniques for the endovascular embolization of vein of galen malformations

**DOI:** 10.3389/fped.2023.1082318

**Published:** 2023-03-23

**Authors:** Jacob F. Baranoski, Joshua S. Catapano, Felipe C. Albuquerque, Todd A. Abruzzo

**Affiliations:** ^1^ Department of Neurological Surgery, Barrow Neurological Institute, Phoenix, AZ, United States; ^2^ Department of Neuroradiology, Phoenix Children’s Hospital, Phoenix, AZ, United States

**Keywords:** vein of galen malformation, embolization, rapid ventricular pacing, arteriovenous malformation, flow control, endovascular treatment, onyx ®, nBCA

## Abstract

Endovascular embolization is the primary strategy in the management for vein of Galen malformations (VOGM). However, despite significant advances in endovascular embolization technologies and techniques, VOGMs remain very technically challenging lesions largely due to the high-flow arteriovenous shunts present in these malformations. A variety of advanced flow-control techniques can be implemented to mitigate the risk of venous escape and increase the safety and efficacy of endovascular treatment. These techniques include regionally targeted strategies (transvenous embolization and balloon-assisted transarterial embolization) and global flow-control methods (pharmacologic cardiac arrest and rapid ventricular overdrive pacing). Each of these strategies are associated with unique advantages and disadvantages, highlighting the importance of a patient-specific approach when treating these challenging lesions.

## 1. Introduction

Vein of Galen malformations (VOGM) are a subset of rare fetal central nervous system (CNS) arteriovenous malformations (AVM) characterized by abnormal development of the primitive choroidal venous circulation and persistence of the median prosencephalic vein (1). In contemporary practice, endovascular embolization forms the cornerstone of VOGM treatment at all ages.

Since the first report of successful endovascular embolization of a VOGM in 1989 (2), endovascular techniques have significantly evolved and endovascular embolization is now universally considered the primary definitive treatment modality for these lesions (1, 3, 4). The technical goal of treatment is effective and durable disconnection of individual high-flow arteriovenous shunts within the VOGM. This typically requires multiple stages of treatment, each addressing one or more individual arteriovenous shunts. A variety of different approaches have been described to treat these dangerous lesions (1, 3, 4).

Despite significant advances in endovascular embolization technologies and techniques – including the development of advanced microcatheter systems and liquid embolysates such as n-butyl cyanoacrylate (n-BCA) glue (Trufill®, Cerenovus, Irvine, CA, USA) and DMSO based ethylene vinyl alcohol co-polymer (Onyx®, Medtronic, Minneapolis, MN, USA), VOGMs remain very technically challenging lesions (1, 3–8). While this is due to number of factors, the most significant is the difficulty associated with successful on-target deposition of embolic material at the fistulous site in the setting of the very high-flow arteriovenous shunt. Both liquid embolysates and detachable coils have poor stability within the high-flow fistulous target. The high-velocity, turbulent flow created by these arteriovenous shunts markedly increases the difficulty of successful, on-target embolization as the torrential background flow washes embolic agent into the venous outflow tract of the fistula, resulting in venous escape of embolic agent into intracranial venous outlets and pulmonary arteries, with potentially catastrophic consequences (1, 3–8).

To increase the safety and efficacy of endovascular treatment for VOGM, several adjunctive flow-control techniques have been developed to counter the challenges created by the extreme high background flow environment (1, 3). All these techniques aim to increase the accuracy of embolization and mitigate venous escape. The means by which these methods achieve this goal vary. Depositing detachable coils within an arteriovenous shunt, through the embolizing microcatheter, prior to administration of liquid embolic is a simple method of flow control that is sometime useful during the embolization of a high-flow arteriovenous shunt. In practice however, coil elongation into the flow stream of the shunt often precludes stable anchoring of the coil. More advanced techniques include the concurrent utilization of transarterial and transvenous embolization (9–13), the use of micro-balloon catheters to suppress arterial flow (14, 15), pharmacologically induced circulatory arrest *via* cardiac pause (16–18), and right ventricular overdrive pacing (19–21). Each technique has advantages and disadvantages and should be employed on a case-dependent basis after careful consideration of specific clinical, anatomical and hemodynamic factors. It is important to consider the highly variable angioarchitecture of these lesions including the number and size of arterial inputs and their relative contributions to the high-flow arteriovenous shunt. Equally important as the technical considerations, a patient-specific approach taking into account the overall fluid status, procedural length of time and radiation exposure, and concomitant risk of heart failure is paramount particularly in neonatal patients. Herein, we present these various techniques and review the available data on their utilization.

### 1.1. Adjunctive flow-control techniques for the endovascular embolization of vein of galen malformations

#### 1.1.1. Transvenous flow-control techniques

Primary transvenous embolization of VOGM has largely fallen out of favor due to the high risk of hemorrhagic stroke resulting from occlusion of normal thalamostriate venous drainage (1, 3, 6–9). Nonetheless, adjunctive transvenous embolization approaches often play an important role in mitigating venous escape during VOGM embolization (9). Some authors have reported partial, non-occlusive coiling of the aneurysmal median prosencephalic vein to “jail” the ostia of choroidal venous tributaries, thus establishing a barrier to prevent venous escape of coils and liquid embolics. This approach can be technically challenging because the sub-occlusive coils may be unstable in the turbulent, high-flow environment of the arterialized vein pouch, and unintended thrombosis of the vein pouch may be precipitated by subsequent transarterial embolization. Additionally, some authors have reported that the interaction of high-flow jets with the intravascular “strainer effect” of the non-occlusive coil matrix can promote hemolysis and disseminated intravascular coagulation (22, 23).To enhance the safety and efficacy of embolization, two transvenous techniques have been developed. These are the “*Brassel kissing microcatheter technique*” (3, 9, 11) and the “*Adapted Chapot pressure cooker technique*” (9, 13).

#### 1.1.2. Brassel kissing microcatheter technique

First described by Meila et al. in 2012 (11), the “kissing microcatheter” technique refers to a combined transarterial and transvenous approach for the treatment of VOGM. With this method, a microcatheter from the venous side is navigated across the arteriovenous shunt and brought into close proximity with an opposing transarterial microcatheter, within the arterial segment of an arteriovenous shunt. The technique leverages the transvenous coil mass to accomplish stabilization of embolic agent delivered through the transarterial microcatheter. The transvenous coil mass is stabilized by the transvenous microcatheter used to deploy it, allowing it to anchor rather than elongate into the powerful flow stream sweeping it into the venous outflow. The obstruction to anterograde migration created by the downstream transvenous microcatheter combines with the push vector of transvenous coil deployment to prevent coil elongation in the high background blood flow. The transvenous microcatheter effectively functions as a “back-stop”. In Meila's original series, complete or near-complete obliteration of the malformation was achieved in 11/14 (78.6%) cases with near 70% achieving an excellent long-term neurological outcome (11).

Advantages of this technique include the customizability of the dual microcatheter setup and the ability to adjust treatment strategy intraprocedurally if the coil embolization is not proceeding as anticipated. Potential challenges include the need for both venous and arterial access and the inherent complications associated with transvenous microcatheter navigation across a high-flow arteriovenous shunt. Multiple access sites can be particularly challenging in this patient population given the overall small caliber and potential fragility of the vessels required to navigate to reach the embolization target. Further, consideration of the possible significant increase in the length of time required to employ these combined transarterial and transvenous embolization techniques warrants consideration – the increased case duration can be associated with increased time of anesthesia as well as increased fluid transfusions both of which can be challenging in this patient population that is prone to potential cardiac complications.

#### 1.1.3. Adapted chapot pressure cooker technique

The “Chapot pressure cooker technique” was originally described as a method for the transarterial embolization of cerebral arteriovenous malformations (24). The technique is centered around creation of an anti-reflux plug in the arterial feeder being embolized. A variation of the technique adapted for VOGM embolization was described by Fifi et al. The method is centered around creation of a “venous escape plug” using a combination of coils, and liquid embolic agent. The venous escape plug establishes venous outflow arrest and promotes retrograde filling of arterial feeders with liquid embolic agent (9, 13, 24).

The adapted Chapot Pressure Cooker Technique for VOGM necessitates at least two venous microcatheters – one detachable tip microcatheter navigated upstream for the delivery of liquid embolic agent (i.e., ethylene vinyl alcohol copolymer), and a second coiling microcatheter placed downstream within the arterialized vein for placement of detachable embolization coils. Coiling through the downstream microcatheter establishes an initial venous outflow arrest and traps the detachable tip of the upstream microcatheter in position for embolization with liquid embolics. This embolizing construct prevents venous escape of liquid embolic agent and promotes retrograde filling of arterial feeders supplying the shunt with liquid embolic (9, 13, 24). Hypotension (< 70 mm Hg) is induced during the embolization of liquid embolic agent. The embolization process is completed by injecting a polymerizing liquid, acrylic (i.e., N-butyl cyanoacrylate) through the coiling microcatheter, prior to extraction of all indwelling devices.

The VOGM-adapted Chapot Pressure Cooker technique is best employed as a final-stage intervention after reducing arterial inflow with multiple rounds of transarterial embolization. Fifi et al. recently described their results using this technique in 4 patients with choroidal type VOGM (9). In their series, the authors achieved complete obliteration of the VOGM with favorable neurological outcome in all four patients. While certainly promising, further studies are warranted to better assess the generalizability of this technique for these challenging lesions.

Advantages of this technique include the ability to achieve a durable cure *via* complete and controlled occlusion of high-flow shunts by primary occlusion of arterialized veins accompanied by secondary occlusion of associated arterial inputs. This technique is technically challenging however and requires multiple transvenous microcatheters in addition to an arterial catheter for control angiography. Further, as Fifi et al. caution, the VOGM-adapted Chapot Pressure Cooker Technique should only be employed after multiple rounds of successful transarterial embolizations (9). Further, care must be taken during the embolization to identify and preserve the deep cerebral venous drainage system, including the thalamostriate and internal cerebral veins. As with the kissing catheter technique, it is important to consider the added risk associated with multiple vessel access sites and the potential complications inherent with an increased length of procedure time and the associated fluid shifts, cardiac stress, and radiation exposure.

### 1.2. Balloon-Assisted embolization

As endovascular techniques continue to evolve, so to do endovascular technologies. In addition to improved microcatheters and liquid embolics, the development of navigable, dual-lumen balloon microcatheters adds another tool to the endovascular armamentarium. In many cases, these balloon microcatheters can be superselectively navigated into the arteries supplying a VOGM. Temporary flow-arrest established by balloon inflation can facilitate on-target delivery of liquid embolic and mitigate venous escape of embolic agent into intracranial venous outlets and pulmonary arteries.

While neuroendovascular balloons, including dual-lumen balloon occlusion catheters, have been available for many years (25), recent technological advances have led to smaller, more flexible and more navigable devices. Such devices can be reliably navigated into and inflated within the extremely tortuous feeding arteries of a VOGM. Dual lumen balloon microcatheters enable delivery of liquid embolic agents through the guidewire lumen of the device while the balloon mounted on the microcatheter shaft is inflated through a separate independent lumen, thus providing flow-control protection as liquid embolic is simultaneously advanced into the target vessel segment. Additionally, multiple balloon occlusion catheters can be used simultaneously to induce temporary flow-arrest in different VOGM feeding arteries converging on the same shunt zone, thus preventing venous washout of liquid embolic agent that reaches the venous segment of the shunt. While most endovascular balloons are compatible with DMSO-based ethylene vinyl alcohol copolymer embolics, the ethiodol in acrylic mixtures has a tendency to promote rupture of endovascular balloons if sufficient surface contact occurs during the embolization process.

One balloon microcatheter suitable for VOGM embolization recently debuted in the United States - the Scepter Mini® (Microvention, Aliso Viejo, CA). White et al. first described the use of this balloon microcatheter for embolization of VOGM (15). In their case report, the authors reported safe and effective utilization of the Scepter Mini® for transarterial embolization of a VOGM. A second case report by Okcesiz et al. reports similar successful outcomes using this approach (14).

While these reports are encouraging and balloon assisted technique has the potential to be advantageous in appropriately selected cases, there are also major risks and limitations to consider. Inflating a balloon in the artery feeding a high-flow shunt can result in catastrophic vessel dissection or rupture. Additionally, prior to flow-arrest, a sub-occlusive balloon can become unstable in the surrounding flow stream, and act as a “sail” to propel the microcatheter “downstream”. Further, while the ability to achieve target flow-arrest is potentially advantageous, it is possible that a balloon inflated in a single arterial feeder will not suppress flow in the venous segment of an arteriovenous shunt when multiple feeders are convergent on the same shunt zone. As noted above, multiple balloon microcatheters can be used simultaneously to address this challenge, albeit this approach has significantly added complexity and risk. Further, the caliber and overall fragility of these vessels presents and inherent challenge when inflating the balloon with potential for inadvertent arterial rupture. While initial reports are encouraging, more studies are of course needed to better assess the efficacy and safety of this technique.

### 1.3. Pharmacological circulatory-arrest / adenosine cardiac pause

In contrast to the anatomically targeted but hemodynamically limited flow-arrest achieved with the use of balloon microcatheters, other adjunctive flow control techniques strive to temporarily induce global blood flow cessation to prevent venous escape of embolic agent.

Adenosine is a purine nucleotide that is the first-line pharmacological treatment for supraventricular tachycardia associated with hemodynamic instability in infants as well as adults (3, 16–18). Adenosine causes hyperpolarization of the sinus and atrioventricular nodes resulting in cardiac standstill. Utilization of “adenosine cardiac pause” to induce temporary circulatory arrest was initially described as an adjunct to open cerebrovascular surgery in the 1980s. Adenosine cardiac pause has more recently been used as an adjunctive method of global circulatory arrest during endovascular procedures, including catheter directed aortic aneurysm repair, coronary artery stenting, and cerebral arteriovenous malformation embolization (3, 16–18).

The appeal of this technique is apparent – temporarily induced global flow arrest allowing for increased accuracy of embolization with the high-flow arteriovenous shunt temporally abated. Indeed some centers have employed this technique to treat challenging VOGMs. Yoon et al. reported three treatments in two patients during which they utilized adenosine cardiac pause for flow control during embolization of VOGMs. The authors found the technique to be a well-tolerated and effective method for flow control during transarterial VOGM embolization (18). Likewise, Tsimpas et al. (17) and Ghorbani et al. (16) reported successful utilization of adenosine cardiac pause for flow control during VOGM embolization.

While the use of adenosine for treatment of arrhythmias, and as an adjunct for flow control during endovascular procedures in children has been shown to be both safe and effective, the half-life of adenosine is extremely short (<10 s), making it difficult to estimate the dose in relation to the timeframe needed for embolization (3, 16–18). The relatively brief and unpredictable protected embolization time window afforded by adenosine cardiac pause can be challenging. A sudden cessation of flow-arrest prior to completion of the embolization process may result in abrupt discharge of embolic agent into the venous outflow of the target arteriovenous shunt. Conversely, extended periods of cardiac standstill may not be well tolerated. Other complications of adenosine infusion include transient atrioventricular block, pulmonary oedema, and bronchospasm (3, 16–18). While pharmacological circulatory arrest may be useful for particularly challenging VOGMs, the technique should be used judiciously given the significant associated risks.

### 1.4. Right ventricular overdrive pacing

Given the benefits of adjunctive pharmacological circulatory arrest during neuroendovascular treatment of high-flow cerebrovascular lesions, other more predictable techniques have been explored to induce global flow reduction. Right ventricular overdrive pacing (RVOP) has been described by multiple authors as an alternative strategy. RVOP reduces cardiac output, decreasing both blood pressure and blood pressure amplitude without producing cardiac arrest (19–21). The hemodynamic effects of RVOP can be accomplished in a markedly more robust and controllable fashion than with pharmacological methods. The main risk of RVOP is that of ventricular dysrhythmia, including ventricular fibrillation.

With RVOP, the right cardiac ventricle is typically transvenously catheterized with a flow directed, balloon tipped, 4 French bipolar pacing catheter or fixed curve quadripolar pacing catheter. Defibrillating pads are placed on the patient's chest as a precaution. The pacing catheter is connected to a temporary external pacemaker (19–21). Just prior to VOGM embolization, RVOP is initiated. The right ventricle is typically paced at a rate of 160 to 250 beats per minute, during which time right ventricular filling is compromised and there is a disruption of atrioventricular synchrony. The process reduces ventricular stroke volume and cardiac output. End-tidal carbon dioxide is used as a non-invasive indicator of cardiac output. In practice, a mean arterial pressure of no more than 30 mm Hg, and a 25%–40% decrease in end-tidal carbon dioxide is targeted. The corresponding reduction in cardiac output leads to a profound decrease in VOGM circulation, enabling embolization with a markedly decreased risk of venous escape. Following embolization, pacing is discontinued and the heart returns to sinus rhythm (19–21). RVOP can safely be extended for periods as long as 60 to 90 s, enabling a wide temporal margin for protected embolization.

**Figure 1:**
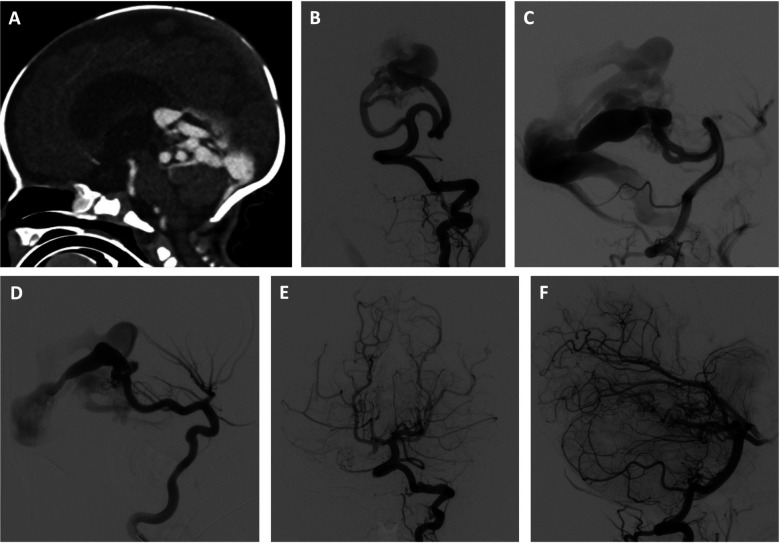
Three-stage embolization of congenital high-flow vermian arteriovenous malformation with acrylic and ethylene vinyl alcohol copolymer using adjunctive right ventricular overdrive pacing for flow control. Newborn male initially presenting with mild neonatal heart failure, which resolved. The patient subsequently experienced failure to thrive and was found to have progressive hydrocephalus. Brain imaging studies revealed a congenital high-flow vermian arteriovenous malformation (AVM) featuring multiple arteriovenous macrofistulae. The patient was treated by two stage transarterial embolization using adjunctive RVOP for flow-control during the first 4 weeks of life and made a dramatic clinical improvement. Use of the RVOP technique was selected for this case given the anticipated extremely rapid transit of embolysate through the high-flow shunt. A transvenous approach was not selected due to the anticipated challenge of navigating a microcatheter through the profoundly dilated venous anatomy. A transarterial balloon-assisted embolization was not selected due to multiple high-flow arterial inputs that could continue to propel the embolysate downstream despite balloon inflation. In the first embolization session, the left superior cerebellar artery supply to the AVM was embolized with a mixture of 80% n-BCA and 20% Ethiodol, and the right posterior cerebral artery supply to the AVM was embolized with a mixture of 90% n-BCA and 10% Ethiodol. An Echelon 10 ® microcatheter (Medtronic, Santa Rosa, CA) was used in both instances. In the second embolization session, embolization of the left superior cerebellar artery contribution was completed using Onyx 34 ® (Medtronic, Santa Rosa, CA), and embolization of of the right superior cerebellar artery supply to the AVM was performed with a mixture of 90% n-BCA and 10% Ethiodol. In both instances, a 1.5 cm detachable tip Apollo ® microcatheter (Medtronic, Santa Rosa, CA) was used. A third stage transarterial Onyx 34 ® (Medtronic, Santa Rosa, CA) embolization was electively performed 6 months later without RVOP, resulting in complete initial angiographic occlusion of the AVM. (**A**) Sagittal reconstruction of CT angiogram shows a congenital high-flow vermian AVM and hydrocephalus resulting from a combination of aqueductal compression and hydrovenous dysfunction. (**B,C**) Early arterial phase of selective left vertebral artery angiogram in frontal (**B**) and lateral (**C**) projections just prior to first stage embolization shows multiple, bilateral mural arteriovenous macrofistulae formed between the superior cerebellar arteries and an aneurysmally dilated superior vermian vein. (**D**) Early arterial phase of selective right internal carotid artery angiogram in the lateral projection just prior to first stage embolization demonstrates the contribution of the right posterior cerebral artery to the AVM. (**E,F**) Late arterial phase of selective left vertebral artery angiogram in frontal (**E**) and lateral (**F**) projections following 3 stages of embolization shows complete initial angiographic occlusion of the AVM.

**Figure 2:**
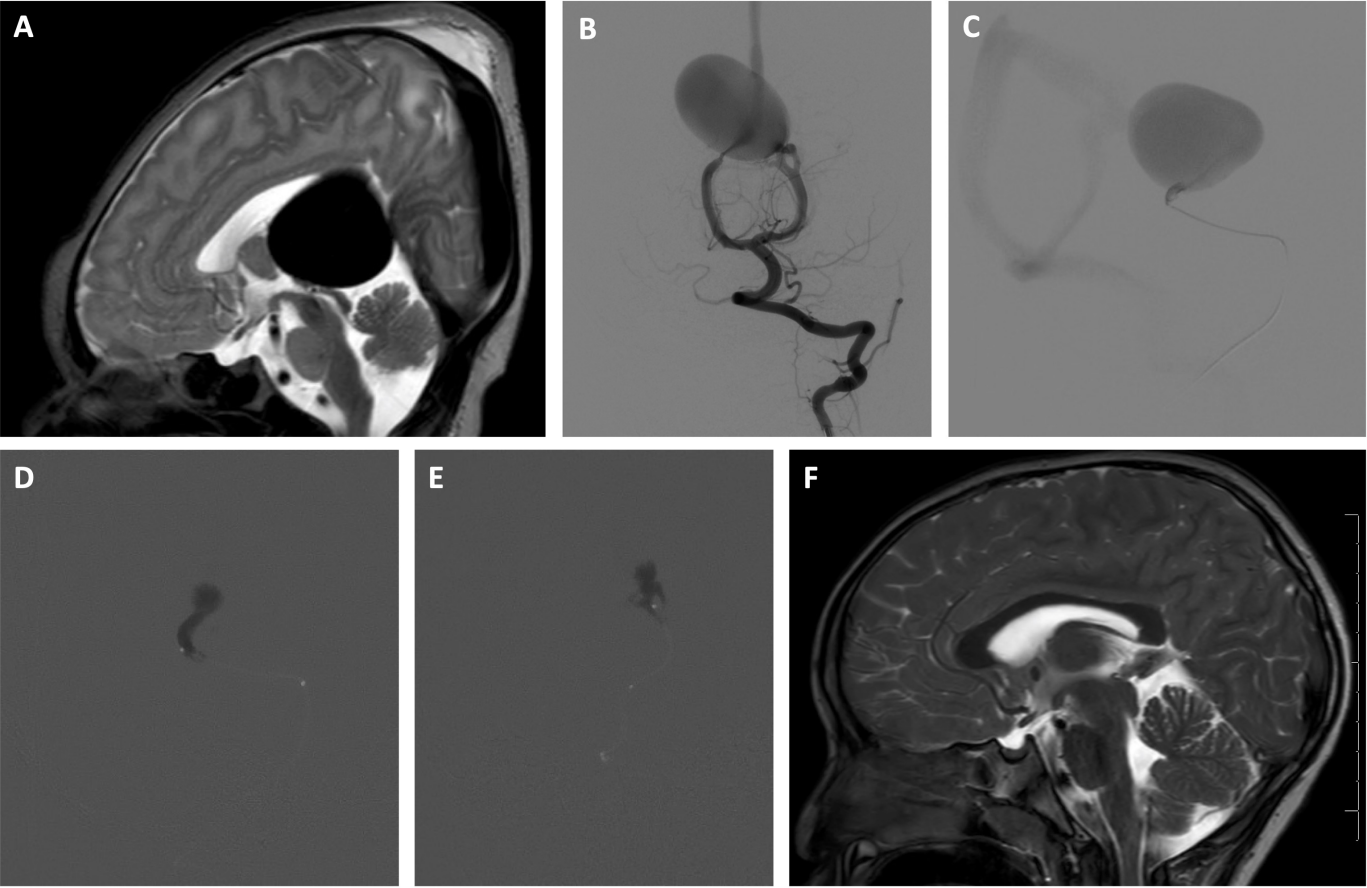
Two-stage embolization of VOGM with n-BCA glue using adjunctive RVOP for flow control. A newborn male, initially found to have a VOGM on prenatal ultrasound performed late in pregnancy because of maternal risk factors was asymptomatic and monitored closely during the first 3 months of life. Use of the RVOP technique was selected for this case given the anticipated rapid transit of embolysate through the hight-flow shunt. A transvenous approach was not selected due to the anticipated challenge of navigating a microcatheter through the profoundly dilated venous pouch. A transarterial balloon-assisted embolization was not selected due to multiple high-flow arterial inputs that could continue to propel the embolysate downstream despite balloon inflation. Utilizing the RVOP adjunctive technique, a two-stage transarterial acrylic embolization with adjunctive right ventricular overdrive pacing was performed resulting in angiographic cure. At three years follow up, the patient is neurologically normal and attending preschool with normal development. (**A**) Sagittal T2 weighted MR image of the brain obtained on day of life 1 shows massively dilated aneurysmal median prosencephalic vein, consistent with vein of Galen malformation. (**B**) Early arterial phase of selective left vertebral artery angiogram in frontal projection just prior to first stage embolization at 3 months of age shows multiple, bilateral mural arteriovenous macrofistulae formed between medial posterior choroidal arteries and aneurysmally dilated median prosencephalic vein (MPV). (**C**) Microcatheter angiogram (lateral projection) of mural arteriovenous shunt supplied by left medial posterior choroidal artery performed through Echelon 10® microcatheter (Medtronic, Santa Rosa, CA) during first stage embolization shows rapid arteriovenous shunting. The microcatheter is stationed just proximal to a sharp bend in the microcatheter bearing artery to reduce the possibility of venous escape. (**D,E**) Lateral (**D**) and frontal (**E**) projections of acrylic cast in the mural arteriovenous shunts supplied by left medial posterior choroidal artery after stage 1 embolization. The embolization was performed during right ventricular overdrive pacing with a 4 French bipolar pacing catheter, pacing at 250 beats per minute for 20 s. Embolization was performed with a mixture of 75% n-BCA and 25% Ethiodol through an Echelon 10® microcatheter. Venous escape of embolic agent was completely suppressed without procedure related complications. Sudden conversion to sluggish flow within the aneurysmal vein (Medtronic, Santa Rosa, CA) pouch prompted anticoagulation for two weeks. Second stage embolization of the mural arteriovenous shunt supplied by the right medial posterior choroidal artery was performed in identical fashion two weeks later without venous escape or other procedure related complications. Complete cerebral angiography performed 3 months after second stage embolization confirmed complete angiographic occlusion of the VOGM. (**F**) At 3 years follow up, sagittal T2 weighted MR image of the brain shows complete involution of the aneurysmal median prosencephalic vein.

RVOP has been used for flow control in adult and pediatric patients during open cerebrovascular surgery and during endovascular treatment of congenital aortic stenosis by balloon aortic valvuloplasty (19–21). Given the successful utilization of RVOP in these types of cases, the technique has been increasingly utilized for flow control during VOGM embolization. The first description of RVOP as a method of flow control during VOGM embolization was first reported by Ramgren et al. in 2017 (21). Our group has successfully utilized adjunctive RVOP for 5 congenital high-flow CNS AVM embolizations in 3 pediatric patients (Figures 1, 2). In all five cases, venous escape of embolic agent was completely suppressed, without procedure-related ventricular dysrhythmia.

Jones et al. (20) recently reported ten cases of RVOP for VOGM embolization. In their series, ventricular capture was achieved in all cases and was well tolerated without induction of arrhythmia. RVOP helped facilitate successful embolization in nine out of the ten VOGMs without any procedural complications (20).

Highlighting some of the potential challenges associated with this technique, Hockley et al. describe their series utilizing RVOP during five embolizations in three VOGM patients (19). In their series, there were two instances when they were unable to achieve RVOP due to loss of myocardial contact of the cardiac pacing catheter. They also reported two instances of induced ventricular fibrillation that were successfully reversed with defibrillation without clinical consequence (19).

There are numerous potential advantages of the RVOP technique to achieve flow-control to facilitate successful embolization of VOGMs. RVOP induces a controlled ventricular tachycardia and the resultant rapid heart rate causes a marked decrease in ventricular filling time and a resultant profound reduction in stroke volume and cardiac output. Employment of this technique can result in abrupt and significant hypotension beyond what can be accomplished with pharmacological means. However, the primary advantage of this technique is that it is also rapidly reversible. The overdrive pacing has a predictable, self-limited response with rapid restoration of normal sinus rhythm upon cessation of pacing. In contrast, adenosine has a variable onset speed and duration of asystole. RVOP provides controllable, transient but sustained hypotension making it ideally suited for facilitating effective embolization of VOGMs.

However, despite these advantages, RVOP is not without its limitations in VOGM patients. As opposed to the adult population, the risks related to RVOP – including iatrogenic ventricular fibrillation – are greater in pediatric patients. Given the other cardiovascular challenges in these high-risk patients, RVOP is not without risk. Therefore, we reserve RVOP for particularly challenging VOGMs with high-flow pedicles that we anticipate or have proven to be challenging for simpler strategies. These challenges also highlight the need for highly specialized, multidisciplinary team including pediatric anesthesiologist and cardiologist during these high-risk procedures. Given the high degree of complexity and risk of the overall procedure, efficient and accurate communication is essential.

## 2. Conclusions

Endovascular embolization forms the cornerstone of management for VOGM. The extreme high-flow nature of these lesions creates extraordinary technical challenges that favor uncontrolled venous escape of embolic agent into cerebral venous outlets and pulmonary arteries. A variety of advanced flow control techniques can be implemented to mitigate the risk of venous escape including regionally targeted and global flow-control methods, each associated with unique advantages and disadvantages. In addition to technical considerations, a multidisciplinary team approach is essential in maximizing the safety and efficacy in these challenging cases. A patient-specific approach selecting the appropriate interventional technique while also considering the overall fluid status, procedural length of time and radiation exposure, and concomitant risk of heart failure is paramount particularly in neonatal patients.
